# How to Improve Value Creation by Service Interaction: The Role of Customer–Environment Fit and Efficacy

**DOI:** 10.3389/fpsyg.2020.01231

**Published:** 2020-06-17

**Authors:** Liang Hong, Hongyan Yu, Tingyi Wang

**Affiliations:** ^1^School of Business, Sun Yat-sen University, Guangzhou, China; ^2^School of Economics and Management, Tsinghua University, Beijing, China

**Keywords:** service interaction, individual–environment fit, value creation, self-efficacy, other-efficacy

## Abstract

Under the service-dominant logic, the interactions between employee and customer create opportunities for value creation. Yet, prior research has ignored the underlying mechanism by which service interaction might improve customer value creation. This study develops a conceptual model of customer–environment fit (C–E fit) from the perspective of customer and conducts empirical research to examine the mediating effect of C–E fit between service interaction and customer value creation and the associated boundary conditions. With data from 435 customer questionnaires, the results show that service interaction has a positive effect on value creation (utilitarian and hedonic); customer–product fit and customer–employee fit act as mediators between service interaction and value creation; customer self-efficacy moderates the mediating effects of two mediators on the relationship between service interaction and value creation; customer other-efficacy only moderates the mediating effects of customer–employee fit on the relationship between service interaction and value creation. Theoretical and practical implications are further discussed.

## Introduction

From new marketing logic, enterprises can co-create value following acceptance of value propositions by customers, but cannot create and/or deliver value independently (Vargo and Lusch, 2008). Enterprises are increasingly aware of the role of customers in value co-creation process and strive to create opportunities to interact with them in the hope of gaining competitive advantages.

As a key in services marketing, interactions have been defined in the concept of service encounter (Lovelock and Wirtz, 2010), which include the interactions between customers and employees, and the interactions between customers and noninterpersonal environment (Shostack, 1985). Bitner et al. (1994) argue that the interactions between customers and employees are particularly important to the evaluation of service satisfaction, which is about information exchange, collaboration, and cooperation (Nardi et al., 2019). In this actor-to-actor exchange system, employees are experts about service and products, and customers are experts in their own lives and conditions (Hau et al., 2017). Then, value is co-created through interaction and resource integration between two sides in the service joint sphere (Grönroos and Voima, 2013). Actor-to-actor interaction takes place to provide service and mutual benefit (Vargo and Lusch, 2008), and it should be understood as service interaction and not simply interfacing or contact (Gummesson and Mele, 2010).

Although the salient role of service interaction in value co-creation has been emphasized (Vargo and Lusch, 2008; Grönroos and Ravald, 2011), interaction is not an automatic shortcut to getting access to customer value creation; instead, it just forms a platform for value creation (Grönroos and Voima, 2013). Moreover, previous research has neglected how service interaction improves customer value creation and the mediating mechanism has not yet been adequately analyzed (Hau et al., 2017), which will obscure the direction of interaction management practices. There are just a few sporadic analyses available (Chan et al., 2010; Yim et al., 2012), except for the perspective of resource integration (Gummesson and Mele, 2010; Koskela-Huotari and Vargo, 2016; Caridà et al., 2018). Then, the interaction between actors can facilitate resource integration through dialogue, resource transfer, and learning. It is worth noting that the guiding principle for resource integration is matching interpreted as the fit between or the consonance of resources, activities and processes, which can further influence value creation (Gummesson and Mele, 2010). However, previous research hardly investigates the notion of fit in the relationships between service interaction and value creation, and suffers from the lack of empirically validated models based on the strong theory.

The concept of fit originates from interactive psychology (Kristof-Brown et al., 2005; Li, 2011; Cheng-Ping, 2018), refers to the interactions between individuals and environment, such as individual–job fit (Tims et al., 2016) and individual–organization fit (Kristof, 1996), and has been widely believed to have important influence on individual attitudes, behaviors, and performance (Kristof-Brown et al., 2005). Although individual–environment fit theory has been adopted by human resource management research (Ostroff et al., 2005; Tims et al., 2016; Shen et al., 2018), there is still a lack of studies grounded in the perspective of the customer. Since customers are often viewed as partial employees in service activities (Dong et al., 2015), individual–environment fit provides the most relevant theoretical approach in the context of service interaction.

To address these issues, we develop a conceptual model based on individual–environment fit theory and conduct empirical research to examine the effect of service interaction on customer value creation and the associated boundary conditions. First, we will propose a conceptual framework of customer–environment fit that explicitly considers the context of service interaction. Second, we will investigate the effect of service interaction impact on the customer value creation. Third, and more importantly, we examine the mediating effect of customer-environment fit between service interaction and customer value creation. Fourth, we present moderated mediation models to examine the moderating effects of customer self-efficacy and other-efficacy on the mediating effects of customer–environment fit on the relationship between service interaction and customer value creation.

## Theoretical Basis and Hypothesis

We use individual–environment fit theory (Kristof, 1996; Tims et al., 2016) as the theoretical basis of our model. The origin of the individual–environment fit theory can be traced back to Parsons’ congruence theory (Parsons, 1909), which is further influenced by Lewins’ social dynamics theory (Lewin, 1945). The former focuses on the relationship between person–organization value congruence and occupational success, and the latter is about organizational socialization and the achievement of person–organization fit. According to the research of individual–environment fit, attitudes and behaviors result from the congruence between characteristics of the person and the environment (Kristof-Brown et al., 2005). The person’s characteristics include individuals’ psychological or biological need, personality, values, or goals; environmental characteristics refer to intrinsic or extrinsic rewards, cultural values, or environmental conditions (Cable and Edwards, 2004).

The individual–environment fit paradigm comprises complementary fit and supplementary fit research; the former focuses on the mode of mutual compensation between person and environment (Kristof, 1996) and the latter is about compatibility between each other (Ostroff et al., 2005). At the individual level, there has been a lot of research on the matching of employees and organizations, leaders, and colleagues (Ostroff et al., 2005; Shen et al., 2018). However, as partial employees and value co-creators, the role of customers and the notion of customer–environment fit have not received the attention it deserves.

**FIGURE 1:**
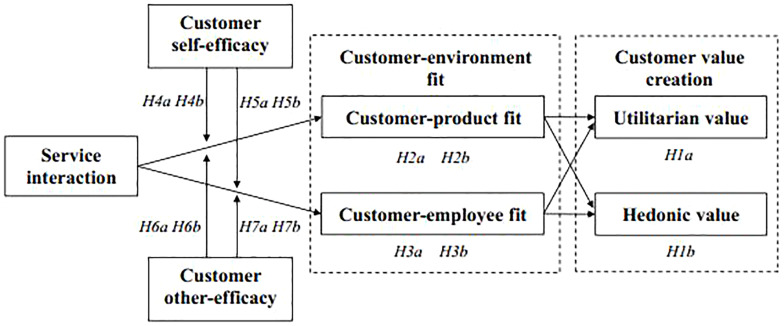
Theoretical framework.

This study constructs the concept of customer–environment fit, which refers to the mutual compensation and compatibility of customer and service environment (Shen et al., 2018), and identifies two forms of customer–environment fit in the context of service interaction: customer–product fit and customer–employee fit. According to the paradigm of complementary fit research (Kristof-Brown et al., 2005), customer-product fit is the degree of congruence between the customer’s preference and the attributes of product or service, which refers to the customer perceiving a match between rewards desired by him or her and those offered by the service provider (Beasley et al., 2012). According to the paradigm of supplementary fit research, the customer–employee fit refers to the compatibility between customer and employee, such as the congruence of their personality, values, and goals (Kristof-Brown et al., 2005). Building on these two fit elements, we argue that good customer–environment fit arises when customers perceive acceptable reward (complementary fit) and perceive compatibility between them and the employees (supplementary fit). Such fit or compatibility would result in positive service outcomes (Kristof-Brown et al., 2005). Figure 1 presents the conceptual framework of this study.

Similarly, Wei and Zhang (2010) argue that customers and employees have their own methods of value creation and evaluation, and their processes are continuously integrated, which may result in the congruence of concepts and behaviors, that can form a system of synergy for value co-creation. Customers do not evaluate service providers, products, or services separately, but rather based on their matching with products, services, and service providers and the significance of these matches (Strandvik et al., 2012; Grönroos and Voima, 2013).

### The Influence of Service Interaction on Customer Value Creation

Grönroos and Ravald (2009) define interaction as “two or more parties are in contact with each other for a commercial reason, and in these contacts they have opportunities to influence one another’ s processes.” In the context of service creation, service interaction emphasizes that two or more parties are in contact with each other in the joint sphere of the service (Hau et al., 2017). Although service interaction can take various patterns (Barros et al., 2005), for the purposes of this study, service interaction refers to face-to-face interactions between customer and employee in a service setting, which takes place to provide service and benefit for the customer. In line with the research of Ivanova-Gongne (2015), we argue that service interaction is an interpretive, communicative, and cognitive process.

According to service-dominant logic, value is co-created mostly through the interactions between service providers and customers in the service joint sphere (Grönroos and Voima, 2013). Hau et al. (2017) emphasize that the core aspects of service interactions are social exchange and information exchange, and these exchanges are the fundamental for service and value co-creation. Consistent with traditional customer value, the co-created value in the joint sphere of the service is multi-dimensional. Previous research on customer participation observed a dual dimension of customer value, with efficiency and usefulness (utilitarian) and enjoyment (hedonic) as the primary benefits of customer participation in service (Rodie and Kleine, 2000; Park and Ha, 2016). This study suggests that the dual dimension of co-created value would hold in a setting of service interaction, in which employee and customer engage in a collaborative relationship to satisfy their needs or wants.

Interactive customers and employees can make decisions on the choice of products or services (Auh et al., 2007), improve service quality, and reduce the risk of failure (Etgar, 2008). Also, high-level customization enhances the efficiency and usefulness of products or services (Chan et al., 2010); these benefits refer to utilitarian value. According to service-dominant logic and value co-creation research, value creation is embedded in experience (Vargo and Lusch, 2008). Some motives of customer participation in service co-creation can be attributed to utilitarian benefits, but also for their own sake and the enjoyable experience (Yim et al., 2012). In the context of service interaction, hedonic value emerges as customer and employee work together in a pleasant and respectful manner (Park and Ha, 2016). Also, these internal states with the rewarding properties of positive affect have been labeled as hedonistic position (Yim et al., 2012). Therefore, we hypothesize the following:

H1a: Service interaction positively affects utilitarian value.

H1b: Service interaction positively affects hedonic value.

### Mediating Effect of Customer–Product Fit

According to the research of service interaction and value co-creation, customer–product fit refers to the fit between customers’ preferences and product (or service) attributes (Moon and Lee, 2014), which is about the relationship between customer needs and offerings (Dong et al., 2015). From a service-centered view, service providers should develop customized and competitive value propositions together with customers to meet their unique needs (Vargo and Lusch, 2004). Similarly, Nätti et al. (2014) argue that service providers can encourage customers to define their needs more concretely, which will be conducive to value co-creation. Because customers generally are more entitled to their preferences than employees, service providers should adopt creative methods to solicit customers’ opinions and suggestions to better embody customers’ idiosyncratic tastes (Dong et al., 2015).

Moreover, some manufacturing firms have positioned themselves as service firms, such as Haier (electric appliance), and they offer numerous design features and options for customization. In a service interaction context, customization can enable customers to choose the features from a set of options that can be supplied by employees. Accordingly, customers can benefit from the interaction when the attributes of customized offerings fit their preferences (Simonson, 2005). Also, customers can take a more active role in adapting offerings, in order to fully benefit from a service (Nätti et al., 2014). For example, customers can make cakes under the guidance of employees to meet their specific needs. In addition, customers with high-level participation can produce better products or services to fit their needs and effectively improve their perceptions of products or services (Dong et al., 2015); such products or services will be more useful and/or cost saving, referring to the utilitarian value.

Furthermore, when the products or services fit the customers’ preferences with their efforts, the customers will consider their efforts to be positive (Franke et al., 2010). As such, the customer will be more engaged, and their sensory experience will be stimulated, which results in positive emotional effects (Benlian, 2015), and these experiences refer to the hedonic value. Thus, we hypothesize the following:

H2a: Customer–product fit mediates the positive relationship between service interaction and utilitarian value.

H2b: Customer–product fit mediates the positive relationship between service interaction and hedonic value.

### Mediating Effect of Customer–Employee Fit

According to the person–environment (P–E) fit paradigm, person–person fit refers to the congruence between personal values and the values of his/her partner, or the compatibility between each other. In the context of service interaction, customer–employee fit refers to the compatibility between customer and employee. We argue that customer–employee fit can be enhanced through the process of service interaction, just as the process of organization socialization that is based on the theory of social dynamics. According to research on person–organization fit, organization socialization can be regarded as the basis for the compatibility between individuals and organization (Kim et al., 2005). Organization socialization is a process of interaction between the person and the organization or group, through which a person can get to know the values and culture of the organization and acquire the necessary skills to work with others (Liu et al., 2013). Similarly, Castanheira (2015) argues that social dynamics would benefit the performance of service interaction.

Compatibility partnership at work means that both parties share common aspects of cognitive processing and common methods of interpreting events, which can reduce ambiguity and conflict (Schein, 1992). Because these common interpersonal feelings and mutual understanding facilitate the predictability of each other’s behaviors and reactions (Price et al., 1995), the information can flow freely among each other (Siegel, 1999). Previous research portrays resource integration as the antecedent of value co-creation (Gummesson and Mele, 2010), and value co-creation requires composite operant resources and interconnected operant resources (Caridà et al., 2018). Thus, customer–employee fit may accelerate the actors’ operation on the available resource and turn a potential resource into a specific benefit. Similarly, Nätti et al. (2014) argue that elucidating can facilitate value co-creation, which means value propositions are based on detailed discussions between the actors, and the aim is to match the customers’ and service providers’ processes.

Customers and employees have many levels of contact; the compatibility between each other can improve the efficiency and effectiveness of service, which is the way to utilitarian value. In contrast, customers tend to experience less comfort when the service provider is cultural incompatibility (Ang et al., 2018). Also, Higgins (2006) argues that the hedonic value comes not only from the outcomes but also from the process. Therefore, if the processes of customers and employees are consistent with each other, customers’ perceived value will be enhanced by the service interactions. Thus, we hypothesize the following:

H3a: Customer–employee fit mediates the positive relationship between service interaction and utilitarian value.

H3b: Customer–employee fit mediates the positive relationship between service interaction and hedonic value.

### The Moderating Effect of Customer Self-Efficacy

Self-efficacy is the “belief in one’s capabilities to organize and execute the courses of action required to produce given attainments” (Bandura, 1977). According to the purpose of the study, we argue that customer self-efficacy is the customer’s belief in his/her capabilities to organize and execute the courses of action required to meet task demands in service interaction; it involves a generative capability by which resources and subskills are orchestrated into successful performance (Gist et al., 1991).

Customers with high self-efficacy are likely to exhibit strong preference recognition abilities and preference expression abilities, and they can effectively improve service quality by the support from the employees. Furthermore, high self-efficacy people tend to engage more, exert more effort, and persist more to overcome task obstacles, and their high self-efficacy prompts them to set challenging goals (Lent and Lopez, 2002; Yim et al., 2012). Thus, customers with high self-efficacy may set high-level goals and strive to achieve them, which means the co-created product or service will be more in line with customers’ requirements.

Previous research has shown that the customers’ satisfaction in production was limited by their abilities (Franke et al., 2009), referring to the preference recognition abilities that customers use to evaluate whether the products that were obtained actually match their needs (Kramer, 2007) and the preference expression abilities that customers use to transmit their preference information to employees (Franke et al., 2009). Thus, customers with low self-efficacy, due to the fact that they do not know exactly what they want, tend to use other cues to build preferences when looking for a specific product. Also, it will be difficult to evaluate whether the offerings actually match their preferences (Kramer, 2007). On the other hand, it is hard for employees to get accurate information through interaction with customers with low self-efficacy, which means the customer–product fit generated by service interaction is inefficient, and the opportunities for value creation might be lost. Therefore, the following hypotheses are proposed:

H4a: Customer self-efficacy moderates the mediating effect of customer–product fit on the relationship between service interaction and utilitarian value, such that the indirect effect of service interaction on utilitarian value via customer–product fit is stronger for higher levels of customer self-efficacy.

H4b: Customer self-efficacy moderates the mediating effect of customer–product fit on the relationship between service interaction and hedonic value, such that the indirect effect of service interaction on hedonic value via customer–product fit is stronger for higher levels of customer self-efficacy.

In order to create high-quality services, customers not only need to know exactly what they want and be good at expressing their needs but also need to have certain knowledge and abilities to get along with their employee partners, to integrate the information and resources of both sides optimally. According to the research of Decety and Meyer (2008), people with high self-efficacy are likely to comprehend the meanings of other people’s words and expressions and the subjective states of others. Then, the customer–employee fit will likely occur during service interactions with customers with a high self-efficacy, which will benefit both parties. On the contrary, customers with low self-efficacy would be ambivalent and confused about the service providers’ motivation (Nijssen et al., 2016) and misunderstand the providers’ service style, resulting in an unpleasant experience.

In addition, self-efficacy refer to the customers’ goal selection, expectations of goal achievement, and persistence in response to difficulties. These beliefs would be important in functioning in personal relationships, in which accommodations are quite necessary and conflict would also be common (Riggio et al., 2013). Thus, customers with high self-efficacy who believe that their behaviors will be effective for collaboration are willing to persist in their relationship in the face of difficulties, which will facilitate the establishment of compatible partnerships and be conducive to value co-creation. Therefore, the following hypotheses are proposed:

H5a: Customer self-efficacy moderates the mediating effect of customer–employee fit on the relationship between service interaction and utilitarian value, such that the indirect effect of service interaction on utilitarian value via customer–employee is stronger for higher levels of customer self-efficacy.

H5b: Customer self-efficacy moderates the mediating effect of customer–employee fit on the relationship between service interaction and hedonic value, such that the indirect effect of service interaction on hedonic value via customer–employee fit is stronger for higher levels of customer self-efficacy.

### The Moderating Effect of Customer Other-Efficacy

Other-efficacy (or proxy control) refers to a person’s beliefs about his or her partner’s abilities to perform particular behaviors, which affects the performance of cooperation not only through the direct abilities of others but also through the degree of an individual’s input (Lent and Lopez, 2002).

Customers with high other-efficacy believe that the employees are competent to identify their preference and provide suitable offerings. Then, customers might not worry too much about the service process (Yim et al., 2012), and they can devote their own abilities and resources to other more valuable activities (Lent and Lopez, 2002), such as seeking more sustainable products or exploring more appropriate approach. Thus, these activities can facilitate the customer–product fit.

Customers with low other-efficacy have low expectations of the outcomes of the collaboration and invest less effort (Lent and Lopez, 2002), which reduces the possibility of accessing high-level service performance. Furthermore, low other-efficacy means that customers perceive more uncertainty about the employees’ perspectives and behaviors. As such uncertainty increases, more cognitive resources will be used for collaboration (Lisa and Juyeon, 2014); then, customers cannot devote their own abilities and resources to other valuable activities. It means that the process of value creation would be impeded. Therefore, we proposed the following hypotheses:

H6a: Customer other-efficacy moderates the mediating effect of customer–product fit on the relationship between service interaction and utilitarian value, such that the indirect effect of service interaction on utilitarian value via customer–product fit is stronger for higher levels of customer other-efficacy.

H6b: Customer other-efficacy moderates the mediating effect of customer–product fit on the relationship between service interaction and hedonic value, such that the indirect effect of service interaction on hedonic value via customer–product fit is stronger for higher levels of customer other-efficacy.

People resort to proxy control when they believe that the others have the ability to help them (Bray et al., 2001); that is, patients are more likely to adhere to health behavior change when they have greater confidence in the expert judgment and therapies of their healthcare providers (Christensen et al., 1996). For the service industry, the abilities of employees to identify and satisfy the customers’ demands are important (Aggarwal et al., 2005). The more accurately the employees understand how the customers receive the service, the more correctly they respond to the customers’ feelings (Gwinner et al., 2005). Furthermore, individuals might experience greater enjoyment and satisfaction with their partners when describing positive other-efficacy (Jackson et al., 2008); thus, other-efficacy can facilitate the compatibility between each other within interactions.

In the context of service interactions, customers with high other-efficacy will prefer to follow the employees’ instructions, which can lead to smooth interactions and clearer information exchanges, and the process will be favorable for both. However, customers with low other-efficacy will be psychologically alienated from the employees, leading to inconsistency of the goals and processes with employees’ goals and processes, which would not be conducive to value co-creation. Therefore, we proposed the following:

H7a: Customer other-efficacy moderates the mediating effect of customer–employee fit on the relationship between service interaction and utilitarian value, such that the indirect effect of service interaction on utilitarian value via customer–employee fit is stronger for higher levels of customer other-efficacy.

H7b: Customer other-efficacy moderates the mediating effect of customer–employee fit on the relationship between service interaction and hedonic value, such that the indirect effect of service interaction on hedonic value via customer–employee fit is stronger for higher levels of customer other-efficacy.

## Method

### Sample and Procedure

The data are obtained from customers’ questionnaires. We required the respondents to recall the service interactions that took place within the last week to ensure that they can still remember the details. Program control and statistical control are used to minimize the common method biases. For program control, constructs were physically separated in the questionnaire, anonymity was provided, and respondents were assured that there are no right or wrong answers (Podsakoff et al., 2003). A total of 513 questionnaires were retrieved from China in 2018, excluding 78 questionnaires that are not carefully answered; thus, we have 435 valid questionnaires, and the rate of effective response is 85%.

The sample consists of 53.56% males and 46.44% females, who are 20 years old and below (15.40%), 21–30 years old (45.52%), 31–40 years old (32.87%), and above 40 years old (6.21%). The respondents’ education background consists of junior college degree and below (25.65%), college degree (37.68%), and postgraduate degree and above (36.67%). The ranges of monthly income cover 3,000 yuan and below (27.13%), 3,001–5,000 yuan (31.49%), and 5,001 yuan or above (41.38%).

### Measures

The original measures were presented in English and then translated into Chinese using standard back translation (Brislin, 1980) for distribution in China. The questionnaires were appropriately modified in accordance with the purpose of the study. All items use a seven-point Likert scale (1 = “strongly disagree,” and 7 = “strongly agree”).

We adopted a behavioral approach to capture the service interaction, and we measured the extent to which the customer interacts with the employee with five items, which include “I spent a lot of time to communicate with the employee,” “I put a lot of effort into expressing my demand information,” “I always provide suggestions to the employee for improving the service outcome,” “employee’s feedback behavior is positive,” and “employee actively solve problems for me” (Chan et al., 2010; Wang and Wan, 2012). Based on the measurement of the fit between the customer and the service/product, we used three items to measure customer–product fit: “The service process looks really great,” “The service offerings come close to my preference,” and “I like the outcome of this service” (Franke et al., 2010; Moon and Lee, 2014). Based on the measurements of person–person fit (Ostroff et al., 2005) and psychological compatibility (Saundersa et al., 1989), the customer–employee fit scale was adjusted slightly to accommodate the context of service interaction with three items: “I agree with the behavior of the employee in the service process,” “The employee understands my feelings very well,” and “The employee has something in common with me.”

We used the customer self-efficacy scale (Yim et al., 2012) to assess customer’s belief in his/her capabilities to organize and execute the courses of action required to meet task demands with four items: “I have confidence in my ability to participate effectively,” “I do not doubt my ability to participate effectively,” “I have excellent participation skills and ability,” and “I am proud of my participation skills and ability.” The customer other-efficacy comprises four items that refer to the customer’s confidence about employee’s service abilities (Yim et al., 2012): “I have confidence in his/her ability to respond to my participation effectively,” “I do not doubt his/her ability to respond to my participation effectively,” “He/She has excellent skills and abilities in responding to my participation,” and “I am proud of his/her skills and ability in responding to my participation.” The utilitarian value scale comprises three items: “convenient,” “economical,” and “quality” (Ryu et al., 2013). The hedonic value scale comprises three items: “enjoyment,” “fun,” and “happiness” (Yim et al., 2012).

## Results

### Reliability and Validity

The results of reliability and validity are presented in Table 1. The Cronbach’s α value of each variable is above 0.70, providing evidence of the reliability (Nunnally, 1978). Measurement validity was tested via confirmatory factor analysis using AMOS17 (Schwepker, 2018). Composite reliability (CR) and average variance extracted (AVE) are often adopted to assess scale validity. The composite reliability of each variable is above 0.8, exceeding a suggested critical value of 0.6 (Bagozzi and Yi, 1988), and each variable’s average variance extracted is above 0.5, exceeding a suggested critical value of 0.5 (Fornell and Larcker, 1981). Thus, the reliability and validity of the measurement constructs meet the requirements.

**TABLE 1:** Reliability and validity of the main measurement constructs.

Variable	Cronbach’s α	CR	AVE
Service interaction	0.834	0.84	0.51
Customer-product fit	0.938	0.94	0.79
Customer-employee fit	0.904	0.89	0.53
Utilitarian value	0.957	0.96	0.85
Hedonic value	0.950	0.94	0.79
Customer self-efficacy	0.941	0.94	0.80
Customer other-efficacy	0.932	0.93	0.78

### Common Method Biases

Although program control is very important to reduce common method biases, statistical control is necessary. We adopted the marker-variable technique and the CFA technique to test the common method biases.

First, we used the marker-variable technique (Lindell and Whitney, 2001) to test the common method biases and take monthly income as a marker variable. Table 2 shows the correlation coefficient matrix of the marker-variable and other variables. The correlation coefficients between the marker-variable and other variables are low and not significant (*p* > 0.05). Thus, the common method biases of this study are not serious.

**TABLE 2:** Correlation coefficient matrix of the marker variable and other variables.

	SI	CPF	CEF	UV	HV	CSE	COE	INCO
SI	1	−	−	−	−	−	−	−
CPF	0.670**	1	−	−	−	−	−	−
CEF	0.600**	0.715**	1	−	−	−	−	−
UV	0.660**	0.857**	0.703**	1	−	−	−	−
HV	0.627**	0.716**	0.680**	0.755**	1	−	−	−
CSE	0.613**	0.465**	0.367**	0.430**	0.489**	1	−	−
COE	0.567**	0.728**	0.661**	0.785**	0.711**	0.368**	1	−
INCO	0.072	0.20	0.055	0.036	0.033	0.049	0.028	1

SI, service interaction; CPF, customer-product fit; CEF, customer-employee fit; UV, utilitarian value; HV, hedonic value; CSE, customer self-efficacy; COE, customer other-efficacy; INCO, monthly income. **Indicates a significant correlation at the 0.01 level (two-tailed test).

Second, we used the CFA technique (Schwepker, 2018; Wei et al., 2018) to test the common method biases. Specifically, there are three steps: (1) All items point to the latent variables measured by them, and carry out CFA analysis, which is called model A. (2) All items point to the common method biases variable, and carry out CFA analysis; this one-factor model is called model B. (3) Compare the changes of model fit indexes of model A and model B if there is significant difference between model A and model B. Based on the results of CFA analysis in Table 3, we find that the model fit of model B is poor, and the chi-square of model A improved significantly (Δχ^2^ = 2902.481, Δ*df* = 22, *p* < 0.001), which means that the common method biases are not serious.

**TABLE 3:** Model fit.

Index	χ^2^	*df*	CFI	NFI	AGFI	RMSEA
Model A	907.453	253	0.943	0.922	0.827	0.077
Model B	3809.934	275	0.690	0.674	0.384	0.172

### Hypothesis Test

#### Main Effect Test

To test the impact of service interaction on utilitarian value and hedonic value, we used SPSS 17.0 software for the regression analysis (Table 4). From Table 4, we can find that service interaction positively influences customers’ utilitarian value and hedonic value. Thus, H1a and H1b are supported. We also find that there was only a slight difference between the standardized regression coefficients of service interaction on utilitarian value (0.655, *p* < 0.001) and hedonic value (0.625, *p* < 0.001), which means that service interaction is equally important to the creation of utilitarian value and hedonic value.

**TABLE 4:** Main effect test.

IV	DV1: utilitarian value				DV2: hedonic value			
	Model 1		Model 2		Model 3		Model 4	
	Beta	VIF	Beta	VIF	Beta	VIF	Beta	VIF
Gender	–0.045	1.019	–0.041	1.019	–0.082	1.019	–0.078	1.019
Age	0.118	1.027	0.027	1.046	0.075	1.027	–0.011	1.046
Education	–0.076	1.008	−0.079*	1.008	−0.127**	1.008	−0.129**	1.008
Service interaction	−	−	0.655***	1.020	−	−	0.625***	1.020
*R*^2^	0.025		0.445		0.032		0.415	
Adjusted *R*^2^	0.018		0.440		0.025		0.410	
*ΔR^2^*	0.025*		0.420***		0.032**		0.383***	
*F*-value	3.648*		86.205***		4.719***		76.290***	
*df1,df2*	(3,431)		(1,430)		(3,431)		(1,430)	

Beta standardized regression coefficient. *p < 0.05 (two-tailed test), **p < 0.01 (two-tailed test), ***p < 0.001 (two-tailed test).

It is interesting to note that the standardized regression coefficients of education on utilitarian value (-0.079, *p* < 0.05) and hedonic value (-0.129, *p* < 0.01) are all significant and negative. The explanation may be that the more educated the customers, the more the perceived cost of time spent in service, and the lower the perceived value from service. Nevertheless, education is not our concern in this study, as are gender and age, which were used as control variables for statistical analysis.

#### Mediating Effect Test

We adopted the bootstrap method to test the mediating effects via the process macro analysis that was developed by Hayes et al. Using Model 4, the number of samples is set to 5,000, the bias-corrected method is used, and the confidence interval is 95%. The independent variable is service interaction, the dependent variables are utilitarian value and hedonic value, the mediating variables are customer–product fit and customer–employee fit, and the control variables are gender, age, and education background. The results are shown in Table 5.

**TABLE 5:** Mediating effect.

Mediation effect	Utilitarian value			Hedonic value		
	Effect	95% confidence interval		Effect	95% confidence interval	
		Lower limit	Upper limit		Lower limit	Upper limit
Overall mediation effect	0.7388	0.6462	0.8392	0.7341	0.6328	0.8500
Customer-product fit	0.5570	0.4559	0.6695	0.5817	0.4872	0.6871
Customer-employee fit	0.1818	0.1073	0.2694	0.1524	0.0831	0.2325

*IV: service interaction, DV: utilitarian value or hedonic value. Bootstrapping based on 5,000 resamples.*

The results show the following: (1) for the relationship of service interaction and utilitarian value, the overall mediating effect of the two mediators is significant, with 95% confidence interval ([0.6462, 0.8392]) excluding zero; the indirect effects of customer–product fit and customer–employee fit are 0.5570 and 0.1818, respectively, with all 95% confidence intervals ([0.4559, 0.6695], [0.1073, 0.2694]) excluding zero, which supports H2a and H3a; (2) for the relationship of service interaction and hedonic value, the overall mediating effect of the two mediators is significant, with 95% confidence interval ([0.6328, 0.8500]) excluding zero; the indirect effects of customer–product fit and customer–employee fit are 0.5817 and 0.1524, respectively, with all 95% confidence intervals ([0.4872, 0.6871], [0.0831, 0.2325]) excluding zero, which supports H2b and H3b.

After controlling the two mediators, the 95% confidence intervals of the direct effects of service interaction on the utilitarian value and hedonic value both exclude zero (utilitarian value [0.0581, 0.2270] and hedonic value [0.0530, 0.2596]). In addition, the direct effect of service interaction on hedonic value is 0.1425 (*p* = 0.0010), and the direct effect of service interaction on utilitarian value is 0.1563 (*p* = 0.0031); these results indicate that the two mediators play partial mediating roles between service interaction and utilitarian value and hedonic value.

#### Moderated Mediation Effect Test: Customer Self-Efficacy

We adopted the bootstrap method to test the moderated mediation effects via the process macro analysis that was developed by Hayes et al. Using Model 7, the number of samples is set to 5,000, the bias-corrected method is used, and the confidence interval is 95%. The results are shown in Table 6.

**TABLE 6:** Analysis of the moderated mediation (self-efficacy).

DV	Indirect effect					Moderated mediation			
	Mediator	Coeff	SE	LLCI	ULCI	INDEX	SE	LLCI	ULCI
UV	CPF	0.586	0.078	0.4581	0.7587	0.045	0.019	0.0094	0.0830
	CPF	0.645	0.077	0.5134	0.8143				
	CPF	0.705	0.083	0.5555	0.8816				
	CEF	0.199	0.047	0.1163	0.3046	0.034	0.010	0.0184	0.0566
	CEF	0.244	0.055	0.1440	0.3617				
	CEF	0.289	0.064	0.1710	0.4271				
HV	CPF	0.612	0.069	0.4848	0.7603	0.047	0.020	0.0093	0.0878
	CPF	0.647	0.069	0.5493	0.8179				
	CPF	0.736	0.078	0.5909	0.8944				
	CEF	0.167	0.043	0.0921	0.2604	0.029	0.009	0.0139	0.0485
	CEF	0.205	0.051	0.1125	0.3117				
	CEF	0.243	0.060	0.1343	0.3677				

*UV, utilitarian value; HV, hedonic value; CPF, customer-product fit; CEF, customer-employee fit.*

As Table 6 shows: (1) Regarding the relationship of service interaction and utilitarian value, the index of the customer–product fit is 0.045 with 95% confidence interval ([0.0094, 0.0830]) excluding zero, which means that there is a significant moderated mediation effect. Additionally, the index of the customer–employee fit is 0.034 with 95% confidence interval ([0.0184, 0.0566]) excluding zero, which means that there is a significant moderated mediation effect. In addition, the indirect effects reveal that as customer self-efficacy increases, the indirect effects of customer–product fit, as well as customer–employee fit, have an increasing trend, with all 95% confidence intervals excluding zero. Therefore, customer self-efficacy has positive moderating effects on the mediating effects of customer–product fit and customer–employee fit between service interaction and utilitarian value. Thus, these results support H4a and H5a. (2) Regarding the relationship of service interaction and hedonic value, the index of the customer–product fit is 0.047 with 95% confidence interval ([0.0093, 0.0878]) excluding zero, which means that there is a significant moderated mediation effect. The index of the customer–employee fit is 0.029 with 95% confidence interval ([0.0139, 0.0485]) excluding zero, which also means that there is a significant moderated mediation effect. In addition, the indirect effects reveal that as customer self-efficacy increases, the indirect effects of customer–product fit, as well as customer–employee fit, have an increasing trend, with all 95% confidence intervals excluding zero. Therefore, customer self-efficacy has positive moderating effects on the mediating effects of customer–product fit and customer–employee fit between service interaction and hedonic value. Thus, these results support H4b and H5b.

**FIGURE 2:**
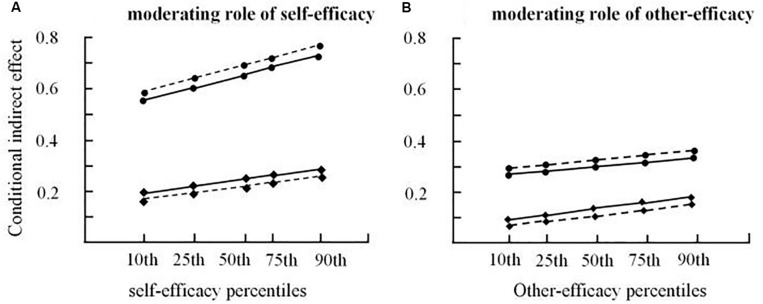
Conditional indirect effect. **(A)** Moderating role of self-efficacy. **(B)** Moderating role of other-efficacy.

Also, we adopted Hayes’ process macro analysis with percentiles conditioning choice to investigate the values of customer self-efficacy and the corresponding indirect effects of service interaction on customer value (utilitarian and hedonic) via customer–product fit and customer–employee fit (see Figure 2). Figure 2A depicts the comparisons of the indirect effects across five customer self-efficacy levels. We find that each indirect effect increases as customer self-efficacy increases, and the indirect effects of service interaction on customer value via customer–product fit are always far higher than the indirect effects of service interaction on customer value via customer–employee fit.

#### Moderating Effect Test: Customer Other-Efficacy

We adopted the bootstrap method to test the moderated mediation effects via the process macro analysis that was developed by Hayes et al. Using Model 7, the number of samples is set to 5,000, the bias-corrected method is used, and the confidence interval is 95%. The results are shown in Table 7.

**TABLE 7:** Analysis of the moderated mediation (other-efficacy).

DV	Indirect effect					Moderated mediation			
		Mediator	Coeff	SE	LLCI	ULCI	INDEX	SE	LLCI	ULCI
UV	CPF	0.287	0.048	0.2015	0.3896	0.022	0.016	–0.0086	0.0545
	CPF	0.318	0.045	0.2349	0.4147				
	CPF	0.348	0.052	0.2530	0.4588				
	CEF	0.078	0.024	0.0403	0.1348	0.018	0.008	0.0037	0.0386
	CEF	0.103	0.024	0.0611	0.1584				
	CEF	0.127	0.029	0.0753	0.1932				
HV	CPF	0.300	0.049	0.2082	0.4005	0.023	0.017	–0.0088	0.0570
	CPF	0.332	0.046	0.2485	0.4305				
	CPF	0.363	0.054	0.2630	0.4763				
	CEF	0.066	0.022	0.0311	0.1151	0.015	0.007	0.0033	0.0335
	CEF	0.086	0.023	0.0454	0.1379				
	CEF	0.106	0.029	0.0565	0.1711				

*UV, utilitarian value; HV, hedonic value; CPF, customer-product fit; CEF, customer-employee fit.*

As Table 7 shows: (1) Regarding the relationship of service interaction and utilitarian value, the index of the customer–product fit is 0.022 with 95% confidence interval ([-0.0086, 0.0545]) including zero, which means that the moderated mediation effect is not significant; the result does not support H6a. The index of the customer–employee fit is 0.018 with 95% confidence interval ([0.0037, 0.0386]) excluding zero, which means that there is a significant moderated mediation effect; the indirect effect reveals that as the customer other-efficacy increases, the indirect effects of the customer–employee fit have an increasing trend. Therefore, customer other-efficacy has a positive moderating effect on the mediating effect of the customer–employee fit between service interaction and utilitarian value. This result supports H7a. (2) Regarding the relationship of service interaction and hedonic value, the index of the customer–product fit is 0.023 with 95% confidence interval ([-0.0088, 0.0570]) including zero, which means that the moderated mediating effect is not significant; the result does not support H6b. The index of the customer–employee fit is 0.015 with 95% confidence interval ([0.0033, 0.0335]) excluding zero, which means that there is a significant moderated mediation effect; the indirect effect reveals that as customer other-efficacy increases, the indirect effects of the customer–employee fit have an increasing trend. Therefore, customer other-efficacy has a positive moderating effect on the mediating effect of the customer–employee fit between service interaction and hedonic value. This result supports H7b.

Figure 2B depicts the comparisons of the indirect effects across five customer other-efficacy levels. We find that each indirect effect increases as customer other-efficacy increases, and the indirect effects of service interaction on customer value via customer–product fit are always higher than the indirect effects of service interaction on customer value via customer–employee fit.

## Discussion and Conclusion

This study finds the following results: First, service interaction has a positive effect on customer value creation (utilitarian and hedonic). Second, customer–environment fit, which comprises customer–product fit and customer–employee fit, mediates the positive relationship between service interaction and customer value creation. The empirical results show that the two mediators (customer–product fit and customer–employee fit) play partial mediating roles. Third, customer self-efficacy and other-efficacy moderate the mediating effect of customer–environment fit on the relationship between service interaction and customer value creation. Specifically, customer self-efficacy moderates the mediating effect of both mediators on the relationship between service interaction and customer value creation; customer other-efficacy only moderates the mediating effect of customer–employee fit on the relationship between service interaction and customer value creation, and it does not work on the mediating effect of customer–product fit.

### Theoretical Implications

In the service industry, customer–employee interaction is deemed a critical aspect to value co-creation, so service providers need to know how to improve value creation by interactions, and what are the guiding principles. Although service interaction and value co-creation have received a high profile in marketing research, the mechanism of service interaction impact on value creation has not yet gotten enough attention. This study is a continuation of resource integration perspective on the relationship of service interaction and value co-creation, which emphasized that the guiding principle for resource integration is matching, interpreted as the fit between or the consonance of resources, activities, and processes (Gummesson and Mele, 2010; Grönroos and Voima, 2013). However, extant research lacks strong theory and empirical analysis. Based on the individual–environment fit theory, we constructed the concept of customer–environment fit, which is grounded in the perspective of the customer in the context of service interaction. It is important, because the positive effect of individual–environment fit on organizational performance has been verified (Kristof-Brown et al., 2005), and customers as partial employees should be concerned in this theoretical framework. Thus, this study builds a bridge between organizational research and marketing research, and extends the research of value co-creation.

In this study, the results verified the key role of fit in linking service interaction to value creation. Such a finding is in line with and qualifies the previous research (Gummesson and Mele, 2010; Grönroos and Voima, 2013; Caridà et al., 2018). As we expected, customer–product fit and customer–employee fit mediated the relationship between service interaction and customer value creation. Although the total two mediators play partial mediating roles, the result is within our acceptance. Because, the notion of customer–environment fit may be richer than our study, other potential fit should be considered. By comparing two mediating effects, we find that the mediating effect of customer–product fit is much stronger than customer–employee fit. There are two possible explanations for this interesting result. First, the primary objective of customer participation is to obtain the desired offerings. Second, the relationship between customer and product (service) might be closer than the relationship between customer and employee, and the information of product (service) is more accessible. The more accessibly and fluently customers process the information of an object, the more positively they respond (Reber et al., 2004).

In this study, we investigated the moderated mediation effect of efficacy, and the results indicate that customers with more confidence in their own and partners’ abilities get more compensation from service and perceive more compatibility with employees, which, in turn, improves the value creation. The results correspond with Grönroos and Voima (2013) research that “it is not resource per se, but the ability to combine them, where customer’s needs, internal linkages, relational goals, networks, and ecosystems all have importance for understanding value creation.” Regardless of the fact that previous studies have emphasized the moderating effects of customer self-efficacy and other-efficacy on the relationship between customer–employee interaction and value creation (Yim et al., 2012; Wang, 2019), there still is a lack of attention to the moderated mediation model. This study fills this gap. Unexpectedly, customer other-efficacy does not moderate the mediating effect of customer–product fit on the relationship between customer–employee interaction and value creation; the possible explanation is that customers’ requirements or goals setting might not depend on the confidence in their partners’ abilities; rather, they focus on their own abilities and resource.

### Managerial Implications

The findings have several implications for firms that are considering or have engaged their customers in value co-creation. First, one could target service interaction to influence customer value co-creation indirectly. Second, one could target customer–environment fit to directly influence customer value co-creation. This could be achieved by fostering the knowledge and the abilities to use customer interaction management strategies adequately. Customers usually define interactions by stating their preferences and needs, and then service providers could co-design products (or services) with them to cater to their experience (Tatiek and Hendar, 2017); thus, customer information acquisition and appropriate feedback are important to the service provider.

Regarding the relationship between service interaction and value creation, customer–environment fit might be a guiding principle for service providers, which comprise customer–product fit and customer–employee fit at least. According to customer–product fit, which refers to the matching between customer preference and offerings, the customer will be proud when such matching is realized by his/her participation, and thus, customer authorization and resource integration by interactive employees’ work would become the premise of value creation. According to customer–employee fit, which refers to the compatibility between their personality, values, and goals, the strategies of traditional customer socialization management might be inadequate. It is difficult to realize compatibility by informative or communicative interactions that actors use to inform others. Then, the strategies of co-creating value propositions might be better (Siltaloppi and Vargo, 2014), which portrays value propositions as constantly evolving.

Furthermore, the moderated mediation process suggests that the indirect effect of customer–environment fit depends on the level of customer self-efficacy and other-efficacy. Customers with rich knowledge and abilities become an important resource for value co-creation, and customer education strategy might be a meaningful attempt to improve customer participation (Yin and Yang, 2009), particularly for customers with low self-efficacy (Theresa et al., 2014). However, the challenge is to identify the level of customer’s knowledge and abilities, and employee’s judgment of customer idiosyncrasy and subsequent behaviors might be impeded. In addition to direct observation, the appropriate CRM strategy embedded in big data technology should be executed, which can obtain multidimensional information from customers. Also, customers’ self-efficacy could be determined and modified by helping them recognize the success of their participation (Yim et al., 2012), and appropriate analyses and encouragements from interactive employees are important.

Regarding the collaboration between customer and employee, the results indicate that value co-creation also depends on the customer other-efficacy, which means that not only the employee’s abilities should be concerned in value co-creation, but also the customer’s beliefs about the employee’s abilities. A person’s other-efficacy could become compensation for his/her own self-efficacy in collaboration activities (Yim et al., 2012). There are several possible tactics to facilitate customer other-efficacy. First, efficacy-related information about employee and organization could be intentionally conveyed to customers, such as certificates, performance awards, and positive comments from other customers. These third-party endorsements might enhance customers’ perceptions of the efficacy of their employee partners (Jackson et al., 2008). Second, employees demonstrating motivation, readiness, and high quality of psychological characteristics (calm or level-headed) could be the cues of their abilities to customers. Finally, the reputation of the firms and employees, such as empathy, warmth, integrity, and conscientiousness, might improve customers’ perceptions of efficacy of their employee partners. Certainly, such reputation can influence partners’ emotional attachment and cooperation security (Chun and Davies, 2010).

### Limitations and Further Research

There are several limitations to this paper that need to be addressed in future research. First, this study develops a conceptual model of customer–environment fit with the primary factors of product, service, and employee. There are also some other environmental factors that should be considered, such as the physical environment. According to vast service research, the quality of the physical environment related to customer perceived value (Ryu et al., 2012). Hence, future research can explore the fit between customers’ personalities and the feature of the physical environment. Second, this study collects data from numerous service industries, and the features of service interactions in different industries are not distinguished. Dong et al. (2015) argued that further segmentation analysis should be conducted to investigate the exact effect of customer participation on service outcomes. Third, the data of this study were collected from customers’ questionnaires; further research can collect matched-pairs data from customers and employees, which not only can reduce some unavoidable bias problems but also can help us better understand service interaction and the process of value co-creation. Fourth, this study focuses on the positive outcome of service interaction, which is customer–environment fit. The negative effect might follow prolonged service interaction; thus, the effort customers put into the service (Franke and Schreier, 2010) should be considered, as well as the nonlinear relationship between service interactions and the outcomes.

## Data Availability Statement

All datasets generated for this study are included in the article/supplementary material.

## Ethics Statement

The studies involving human participants were reviewed and approved by Sun Yat-sen Business School. Written informed consent for participation was not required for this study in accordance with the national legislation and the institutional requirements.

## Author Contributions

LH and HY worked on the theoretical framework, methodology, data collection, and results analysis in equal measure. LH, HY, and TW worked on manuscript writing, and manuscript revising. All authors contributed to the manuscript and approved the submitted version.

## Conflict of Interest

The authors declare that the research was conducted in the absence of any commercial or financial relationships that could be construed as a potential conflict of interest.
